# Effects of Recycled Polyethylene on Natural Rubber Composite Blends Filled with Aluminum Trihydroxide and Polyurethane Waste: Mechanical and Dynamic Mechanical Properties, Flammability

**DOI:** 10.3390/polym16121657

**Published:** 2024-06-11

**Authors:** Varanya Tilokavichai, Onanong Pannarungsee, Piyawadee Luangchuang, Yeampon Nakaramontri

**Affiliations:** 1 Innovative Entrepreneurship Management, Graduate School of Management and Innovation, King Mongkut’s University of Technology Thonburi, Bangkok 10140, Thailand; 2 Sustainable Polymer & Innovative Composite Materials Research Group, Department of Chemistry, Faculty of Science, King Mongkut’s University of Technology Thonburi, Bangkok 10140, Thailand; onanong.pann@kmutt.ac.th (O.P.); piyawadee232800@gmail.com (P.L.)

**Keywords:** natural rubber, polyethylene waste, polyurethane waste, flammability, silica

## Abstract

This research studies natural rubber (NR) composite blends prepared with recycled polyethylene (PE), polyurethane waste (PU), silica (SiO_2_), and aluminum trihydroxide (ATH) under the proper mixing conditions using an internal mixer and a two-roll mill. The mechanical, impact, dynamic mechanical, and thermal properties, together with flammability, were investigated. NR/PU composites filled with a specific SiO_2_/ATH concentration resulted in excellent flame-retardant properties without using PE. Adding PE causes poor flammability, while using PU and SiO_2_ prevents flame extensibility of the composites. In addition, SiO_2_ and ATH synergistically improved both mechanical and dynamical mechanical properties. This is attributed to the reinforcement of SiO_2_ particles inside the matrix, whereas the ATH releases water as a flame retardant. The V-0 composites tested with UL-94 showed acceptable heat resistance, strength, and durability, making them suitable for interior and exterior applications in buildings without the lightweight requirement.

## 1. Introduction

Currently, widespread pollution and contamination from plastic waste directly impact living organisms, particularly the respiratory system. Using recycled plastic is a key issue aligned with the sustainable development goals (SDGs). However, recycling plastic through multiple heating processes to produce replacement products degrades the properties of the original material due to the loss of molecular chain integrity of the plastic, making it difficult to control the quality. Consequently, recycled plastic products are not favored.

Hence, research into and development of recycled plastic materials to create new consumer-oriented, durable, and long-lasting products to replace disposable ones have become a topic of interest. The most promising new recycled plastic products that could change the market are polymer blends created by mixing recycled plastic waste with flexible materials, such as natural rubber (NR), which has various properties and can enhance plastic properties. This blend of plastic and NR is called thermoplastic NR (TPNR), which combines the hardness of plastic with the flexibility and impact resistance of NR. Examples of thermoplastic materials are polyethylene terephthalate (PET), polypropylene (PP), and polyethylene (PE).

PE is one of the most widely used plastics in packaging manufacturing. PE exhibits the highest flexibility among the polyolefins. The mechanical properties of PE include low stiffness, low resistance to creep, low tensile strength, good impact resistance, and poor flammability. In contrast, NR has been extensively studied due to its widespread use and boasts excellent tensile strength and high elasticity. Typically, thermoplastics are blended with NR to facilitate convenience in manufacturing processes by reducing melt viscosity and improving performance [1,2]. This causes superior resistance to heat aging and ozone resistance compared to pure NR and PE [3]. In particular, low-density (LDPE) and high-density PE (HDPE) combined with NR can produce high-quality lightweight microcellular shoe sole products [4] and roofing materials [5]. In addition, blending with NR can reduce the product cost of TPNR, and it is environmentally friendly.

Incorporating other plastic waste, i.e., polyurethane (PU), into plastic and NR products to gain other functional properties has been explored; the resulting structural properties and characteristics depended on the urethane groups [6]. PU exhibits excellent tear resistance, high elasticity, transparency, excellent abrasion and thermal resistance, and good absorption of impact forces. It is resistant to oxygen, ozone, sunlight, oil, solvents, and fats [7,8]. Despite several advantages, it has poor thermal stability and this limitation of PU could be overcome by preparation of the PU nanocomposites filled with several types of fillers under various concentrations [9,10,11,12,13,14,15,16,17,18], and also blending with other compatible and incompatible/compatibilizer composites [19,20,21,22,23]. As a result, the combination of NR and waste PE and PU can improve the intrinsic properties of the resulting materials.

Flammability can be effectively reduced by adding a flame retardant, which slows the spread of fire by slowing down flame propagation and inhibiting smoke generation. Flame protection can occur through two mechanisms: (i) halogen and (ii) halogen-free. Wang et al. [24] have reported the role of double-layered co-microencapsulated ammonium polyphosphate and mesoporous (MCM-41) in intumescent flame-retardant NR composites, resulting in V-0 in the UL-94 flammability test, together with the ammonium polyphosphates (APP), with an MCM-41:APP of 39:1 phr. Khanlari and Kokabi [25] have studied the effect of organically modified montmorillonite (org-MMT) on the thermal characteristics and flame retardancy of NR, as well as the hardness and mechanical properties. Adding 3 wt% org-MMT decreased the aging hardness by more than 55% and delayed the ignition time by about 150%. The heat release rate peak value was reduced by 54% compared to pure NR. Radhakrishnan and Gopinathan [26] have studied the thermal stability of polyvinyl chloride (PVC) and NR-grafted–PU copolymer blends. It was found that the thermal stability and resistance of the blend increased with increasing the block copolymer loadings. This reduced significantly the molecular chains degradation, especially the hydrocarbon chains in PU molecules, which had a maximum temperature of approximately 180 °C [27,28,29,30].

Guler et al. [31] have used aluminum trihydroxide (ATH) and magnesium hydroxide (MH) to reduce flammability. These use the two main mechanisms to delay the combustion process, including the dilution of volatile gases and catalytic effects, leading to enhanced flame retardancy [32]. In the case of ATH, water release occurs at around 220 °C, while MH releases water at around 330 °C. Thus, using ATH in NR composites offers several advantages, including low cost, non-toxicity, and excellent flame retardancy [33]. Combining NR with ATH and flame-retarding filler and waste is promising but challenging because no prior productive formulation can minimize the consumption cost of the product.

Therefore, the present work prepares NR composites filled with SiO_2_ and PU waste with and without blended PE waste to study the mechanical and dynamic mechanical properties related to thermal resistance and flammability before and after the addition of ATH flame retardant. The standard test of UL-94 was used, and the blending concentration (over 100 phr) was chosen using the purposed-on weight and cost reduction to propose the application as both interior and exterior applications. In addition, a high concentration of the the wastes aims to prepare the value-added NR composites, replacing the elimination of those polymeric wastes via combustion processes and also strong reducing of the microplastic to nature.

## 2. Experimental and Characterization

### 2.1. Materials

Natural rubber (NR) (Standard Thai Rubber 5L, STR5L) was supplied by Geefin Rubber Tech Co., Ltd. (Songkhla, Thailand) with a molecular weight higher than 10^6^ g/mol. Aluminium trihydroxide (ATH) with a purity of 99.6% and an average particle size of approximately 10 μm, and the silica (SiO_2_) with the diameter ranges of 10–50 nm were purchased from Bos Ofticon Co., Ltd. (Songkhla, Thailand). In addition, polyethylene waste (PE) was commercially obtained from Union Petrochemical Public Co., Ltd. (Bangkok, Thailand) following the density of 0.949 g/cm^3^ together with an average flow rate of 0.05916 g/min, relating the high-density PE waste. In the case of PU waste, it was a powder with approximately 250 μm of particle sizes obtained from the thermal insulative PU foam. This was received from P.U. Foam Insulation and Trading Co., Ltd. (Samut Prakan, Thailand). Activators and plasticizer were commercially provided by Imperial Chemical Co., Ltd. (London, UK). Accelerator (MBTS) and antidegradant (TMQ) were commercially provided by Flexsys Inc., (Termoli, Italy). Sulfur was commercially provided by Ajax Chemical Co., Ltd. (Samut Prakarn, Thailand). All chemicals and formulations are summarized in Table 1.

### 2.2. Preparation of NR/PE Composites Filled with PU Waste, SiO_2_, and ATH

The NR and PE wastes and their blend composites filled with PU waste, SiO_2_, and ATH were formulated with NR:PE ratios of 100/0, 50/50, 0/100 and mixed using an internal mixer (Model MX75; Charoen tut Co., Ltd. Samutprakarn, Thailand) at a temperature of 150 °C, rotor speed of 60 rpm, and a fill factor of 75% for the PE compound (PEC) and the NR and PE waste blends. The NR compound (NRC) was fabricated using the internal mixer for the composites without PE, but the mixing operation was performed at 80 °C and 60 rpm. The mixing process can be divided into three main parts as follows:

**Part 1:** The NRC was prepared by masticating the NR for approximately 3 min at 80 °C to reduce its molecular weight and surface tension before other chemicals were added, including the activators, PU waste + SiO_2_, ATH, antidegradant, accelerator, and sulfur. With a controlled total mixing time of 19 min, the NRC was dumped and passed on the two-roll mill several times to improve the dispersion and distribution of the fillers inside the bulk NR matrix. For the NR composite, the NRC was pressed through the compression molder at 160 °C under pressure and a fixed compressing time of 15 min. This sample is referred to as NRC-ATH-PuSiO_2_.

**Part 2:** The PEC was prepared by masticating at 150 °C until the PE waste was melted. Then, the PU waste + SiO_2_ and ATH were added to the molten PE and the mixing continued for a total mixing time of 15 min before the PEC was released. The compound was crushed into a small piece to produce the PEC sheet and eventually compressed using the compression mold: 5 min of preheating, another 5 min of compression, and 20 min of cooling. The compound is referred to as PEC-ATH-PuSiO_2_.

**Part 3:** The TPNR was prepared by blending NRC-ATH-PuSiO_2_ with pure PE waste and with PEC-ATH-PuSiO_2_ at 50:50 phr, which are referred to as NRC-ATH-PuSiO_2_/PEC and NRC/PEC-(ATH-PuSiO_2_), respectively. The mixing was performed using an internal mixer at 150 °C by melting the pure PE and PEC-ATH-PuSiO_2_ for approximately 5 min. Then, the NRC-ATH-PuSiO_2_ (without compression) was added before the mixing was continued and the NR was vulcanized.

## 3. Characterization

### 3.1. Mechanical Properties

The dumbbell-shaped tensile test specimens were punched from a molded sheet. The tensile properties were tested based on ISO 37:2011 [34] using a Zwick Z1545 Universal testing machine (UTM; Zwick GmbH & Co. KG, Ulm, Germany) under ambient conditions (25 ± 2 °C). The Young’s modulus, tensile strength, and elongation at break of the composites were measured at an extension speed of 200 mm/min using reciprocal specimens. In addition, the hardness of each composite was measured using a hardness (Shore A) tester following ASTM D2240-05 [35]. It is noted that the five duplicate samples were used for each formulation.

### 3.2. Impact Strength

The notched Izod impact strength of the composites was measured at room temperature following ASTM D256 [36] with a 453 g (1.0 lb) and 63.5 × 12.7 × 10 mm^3^ pendulum volume. The samples were notched to a depth of 1.5 mm and a radius of 0.25 mm along the thickness direction. The tests were performed using a vertical cantilever beam, and the samples were impacted on the notched face by a single swing of the pendulum, causing the crack to propagate from the tip of the notch. A minimum of three specimens were tested for each composition.

### 3.3. Dynamic Mechanical Properties

The dynamic mechanical properties of the composite were tested using a dynamic mechanical analyzer (DMA) in the three-point bending mode, conducted over a temperature range of −150 to 100 °C at a frequency of 1 Hz with a heating rate of 5 °C/min.

### 3.4. Thermal Properties

Heat resistance analysis was performed to determine the thermal resistance of the composite using a thermogravimetric analyzer (TGA). The test was conducted from 30 to 900 °C at a heating rate of 10 °C/min. A nitrogen (N_2_) atmosphere was used from 30 to 600 °C and switched to an oxygen (O_2_) atmosphere from 600 to 900 °C.

### 3.5. UL-94 Flammability Standard Test

**Figure 1:**
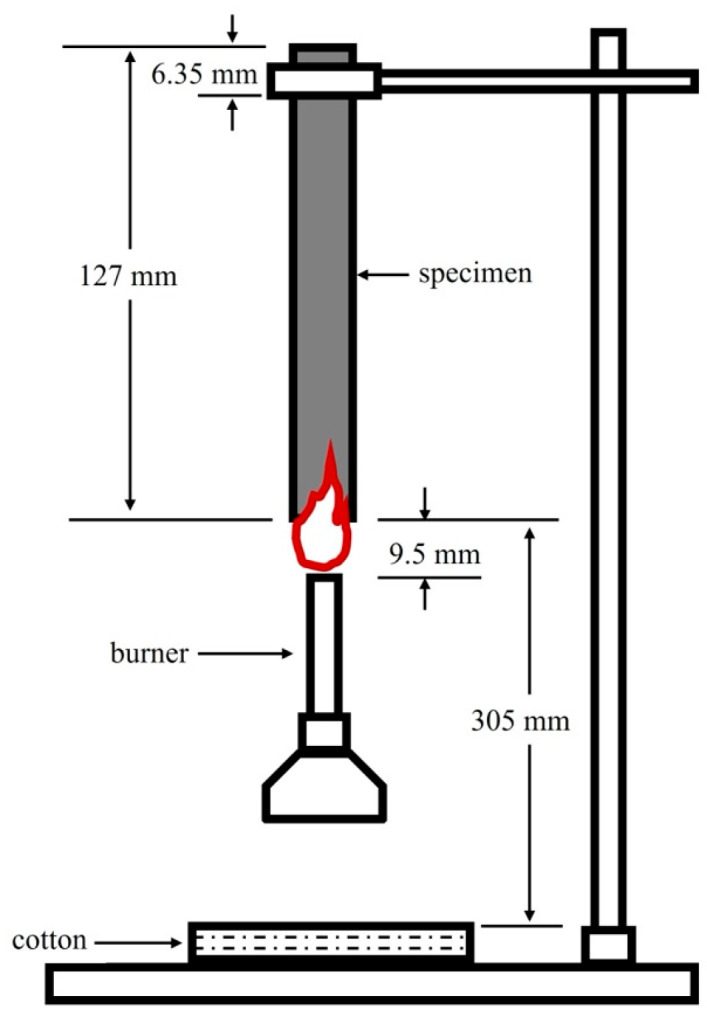
The scheme for the UL-94 vertical burning test [39].

The UL-94 flammability test is performed according to the placement state of the sample during testing. This test is subject to an international standard (IEC 60695-11-10 [37]) and can be divided into horizontal and vertical burning tests. The vertical burning test is generally applied to measure flame retardancy, as shown in Figure 1. In the UL-94 vertical burning test, the burn ratings are classified as V-0 (the flame is extinguished within 10 s), V-1 (the flame is extinguished within 30 s, and unable to ignite the cotton wool located 30 cm below), V-2 (the flame is extinguished within 30 s, and able to ignite the cotton wool located 30 cm below), and HB (the lowest fire rating in the UL-94 flammability standard) [38].

## 4. Results and Discussion

### 4.1. Mixing Torque

**Figure 2:**
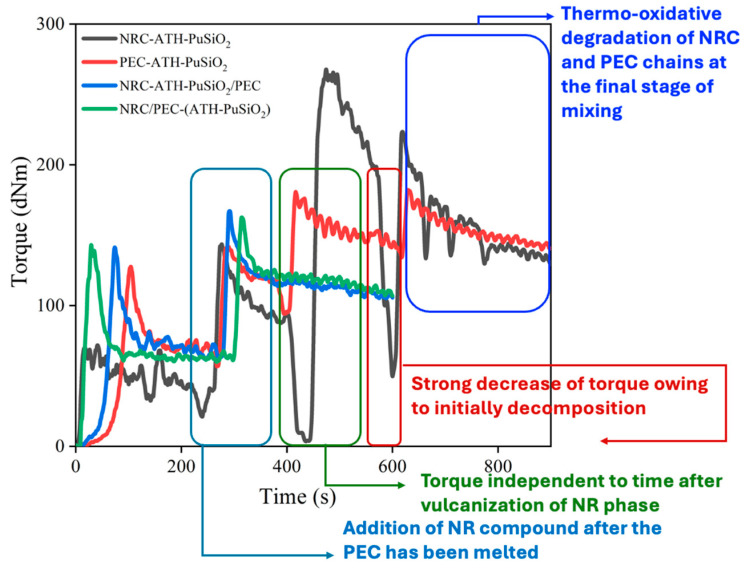
Mixing torque operation of the blending NRC-ATH-PuSiO_2_ with pure PE waste and with PEC-ATH-PuSiO_2_ at 50:50 phr for the preparation of thermoplastic vulcanizates.

Figure 2 shows the mixing torque during the mixing operation of the components as a function of time. It is noted that the first increase and decrease of the torque is due to the molecular chains scission of NR (black line) and the melting of PE before keeping constant for approximately 3 min (i.e., blue, red, and green lines). Then, the torque was again increased due to the addition of the NR compound. The rubber phase was masticated and continuously vulcanized. The torque was kept as constant for another 2 min for decreasing the vulcanized NR particles. Finally, without the decreasing of torque, the compounds were removed from the mixer and compressed into a sheet under the controlled compressing conditions described in Part 2. Thus, regarding the lack of increment of the mixing torque during high temperatures, there was no chemical interaction among the NR-PE, NR-PE-SiO_2_, and NR-PE-PU, while the physical intermolecular forces are encouraged due to dispersion forces among the hydrocarbon molecules of NR and PE, the induced dipole–dipole interaction regarding the NR and PE with SiO_2_ and with PU, and the dipole–dipole attraction following the polar functional groups of the SiO_2_ and PU surfaces. On the other hand, regarding the thermo-oxidative degradation of NR molecules during the mixing operation, the higher optimal mixing torque compared to the initial torques elucidates the predominant reinforcement mechanism relative to mechanical and dynamic chain scissions.

### 4.2. Mechanical Properties

**Figure 3:**
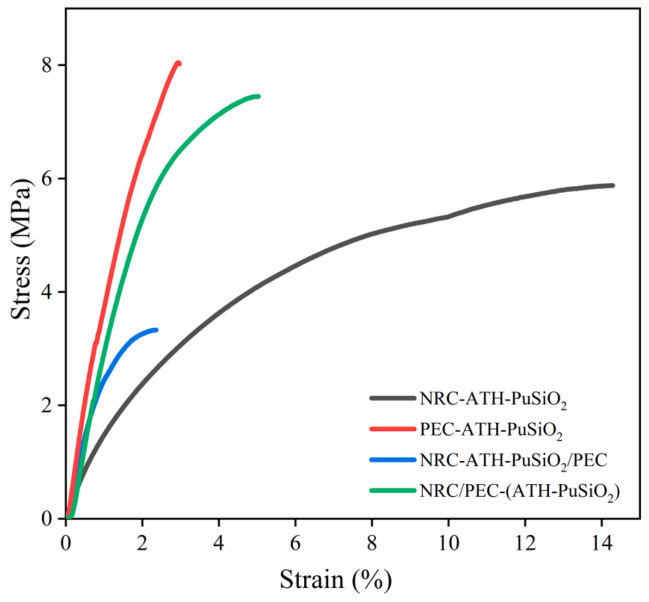
Stress–strain curves of NRC and PEC filled with ATH and PuSiO_2_ under different mixing processes.

The Young’s modulus, tensile strength, and elongation at break from the stress–strain curves are reported in Figure 3 and summarized with the hardness in Table 2. Figure 3 shows that the NR showed very low toughness and strain-induced crystallization degree (very low stress–strain curve increment before the chains were broken), regarding the estimated stress–strain relation areas, due to the high concentration of filler inside the bulk NR, PE, and NR/PE matrices. However, combining NR with the composites increased the strain ability, while adding the PE enhanced the stiffness based on the Young’s modulus of the composites. In Table 2, NRC-ATH-PuSiO_2_ has a tensile strength of 5.87 MPa, which is lower than that of PEC-ATH-PuSiO_2_ (8.02 MPa). This is because the formulations containing PE as a main component have a short-chain hard-segment molecular structure, which gives a higher Young’s modulus and tensile strength due to the retardation of deformation. Additionally, the PU waste and SiO_2_ reinforced the PE and NR phases due to melt mixing. However, the highest viscosity of NR compared to other non-crosslink polymers might cause both fillers to mix less well and agglomerate in the main matrix. The incompatibility of both phases provided defect points at the rubber–filler interfaces, and the tensile strength of the NRC-ATH-PuSiO_2_ decreased.

The NR/PE blends, i.e., NRC-ATH-PuSiO_2_/PEC and NRC/PEC-(ATH-PuSiO_2_), are 50:50 phr blends of NRC with pure PE and PEC-ATH-PuSiO_2_, respectively. The combination of NRC with PEC-ATH-PuSiO_2_ showed higher mechanical properties than the combination of NRC with pure PE, showing that the reinforcement efficiency of the blends can be effectively obtained by incorporating them. These additional fillers have greater potential for dispersion and distribution throughout the NR/PE matrix. As expected, NRC showed the highest values for the elongation at break, whereas the PEC had the lowest, and the NRC blended with PEC presented a higher value than the blend with pure PE. Table 2 shows the hardness of NRC and PEC and their blends, which are well-known to strongly relate to the hard phase of the PE waste at room temperature. Thus, PEC showed the highest hardness, while the high reinforcement efficiency of NRC/PEC-(ATH-PuSiO_2_) produced a higher hardness than in NRC-ATH-PuSiO_2_/PEC. Therefore, from the mechanical properties, the presence of NR and PE provided improved extensibility and tensile deformation resistance, respectively. The blend of NRC and PEC has a higher potential than that of pure PE for TPNR.

### 4.3. Impact Strength

**Figure 4:**
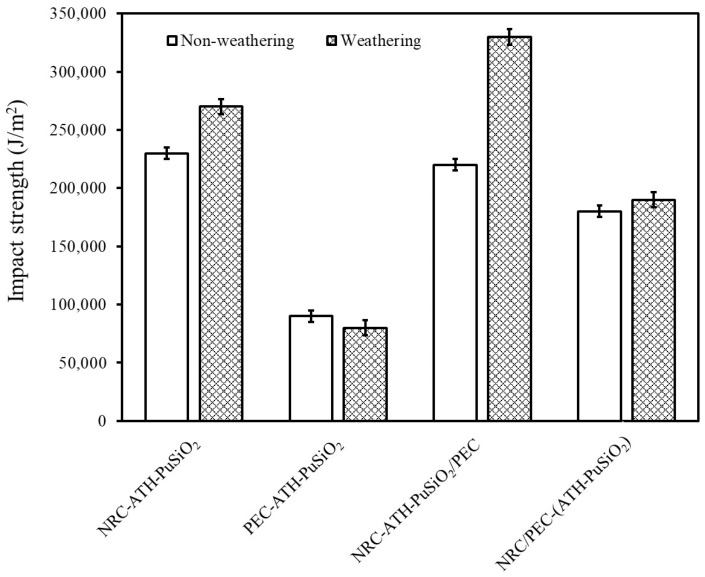
Impact strength of NRC and PEC filled with ATH and PuSiO_2_ under different mixing processes.

Figure 4 shows the impact strength of NRC and PEC filled with ATH and PuSiO_2_ composites before and after a weathering simulation. Without weathering, NRC-ATH-PuSiO_2_ exhibits the highest impact strength compared to the other samples because NR has high elasticity and excellent impact resistance. However, after PE waste was added, the impact strength decreased due to the intrinsic rapid cracking propagation of PE, which produced high stiffness with low toughness. This is why PEC-ATH-PuSiO_2_ had the lowest impact strength compared to the other samples that contained NR. In addition, when the composites and blends were examined after the weathering tests, the impact strength increased slightly relative to the unweathered samples due to the accelerated aging phenomena. This effect induced chemical crosslinking inside the NRC component after chemical crosslinks were generated between sulfur radicals and the remaining allylic carbon and C=C double bonds. PEC-ATH-PuSiO_2_ showed differing behavior because crosslinking was not accelerated between the PE molecular chains. In Figure 4, NRC-ATH-PuSiO_2_/PEC shows the highest impact strength after weathering, which is related to the increased density of crosslinking in NRC particles inside the PEC matrix. This causes expansion throughout the matrix and resistance to breakage under impact testing. Relative to NRC-ATH-PuSiO_2_, the lower NR concentration with the agglomeration of the ATH and PuSiO_2_ might prevent aging acceleration and lowered the impact resistance.

### 4.4. Dynamic Mechanical Properties

**Figure 5:**
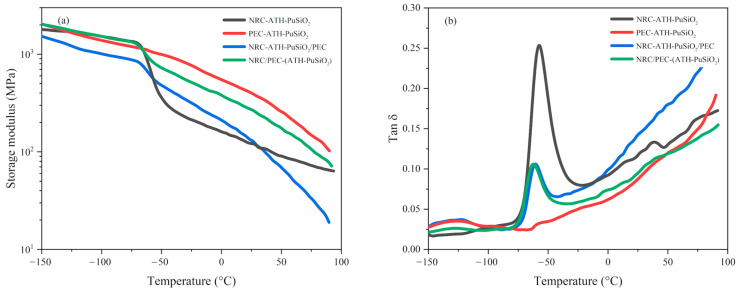
Indication of the *E’* (**a**) and *Tan δ* (**b**) of NRC and PEC filled with ATH and PuSiO_2_ under different mixing processes.

Figure 5 shows the storage modulus (*E’*) and tan delta (*Tan δ*) as a function of the temperature of the composites and blends at chemical concentration together with the values summarized in Table 3. *E’* measures the maximum energy stored in the material, providing valuable insight into the stiffness and elasticity of the composites. When temperature increases, the components become more mobile; hence, a rapid decrease in *E’* called the glass transition temperature (*T_g_*) occurs. In Figure 5a, the initial *E’* correlated to the moduli of the specimen, which was directly related to the existing PE waste component inside the bulk composites and blends. Thus, the highest of the initial *E’* were obtained from PEC-ATH-PuSiO_2_ and NRC/PEC-(ATH-PuSiO_2_) due to the crystalline phases of PE. However, NRC-ATH-PuSiO_2_/PEC showed the lowest initial *E’* due to the incompatibility of NRC and pure PE, correlating to the measured mechanical properties of the blends. Focusing on the role of NRC in the appearance of *T_g_* following *T_g1_* and *T_g2_,* which refer to the *T_g_* of PEC and NRC, respectively, PEC had a lower *T_g_* than NRC; thus, decreasing the amount of PEC and increasing NRC tended to increase the *T_g_*. Based on the *T_g2_*, the combination with PEC lowered *T_g_* due to the additional PEC in the blends. The findings exhibited strong correlation with those of other thermoplastic elastomers across various polymeric blends, including PE/NR [40], PP/NR [41], polycaprolactone (PCL)/NR [42], and nylon (PA6)/acrylonitrile rubber (NBR) [43].

Regarding *Tan δ* at 0 °C versus temperature, as shown in Figure 5b and Table 3, which was used to measure the ratio of the loss modulus (*E’’*) and *E’* of the specimens, *Tan δ* at 0 °C showed different results after the addition of filler and blending with PE waste. PEC-ATH-PuSiO_2_ showed the lowest *T_g_* of the PEC blends, and the lowest *Tan δ* at 0 °C was due to the high *E’* with a low *E’’*. The *Tan δ* at 0 °C of NRC-ATH-PuSiO_2_ was lower after blending with PEC. In the case of NRC/PEC-(ATH-PuSiO_2_), it was lower because the polymeric phases of NRC-ATH-PuSiO_2_/PEC had poor compatibility. Considering the peak height of the *Tan δ*, which denotes the resistance changes from the glassy to rubbery states of the material, it escalates with the increasing elasticity of the material. As illustrated in Figure 5b, the highest peak height is attributed to the elasticity of NRC, which correlates with its extendability as indicated in Table 2, while the lowest value is demonstrated in PEC, characterized by poor elasticity. In the case of NRC and PEC blends, no significant differences were observed, indicating that both blend processes exhibit comparable resistance to the transition from the glassy state to rubbery characteristics.

### 4.5. Thermal Properties

**Figure 6:**
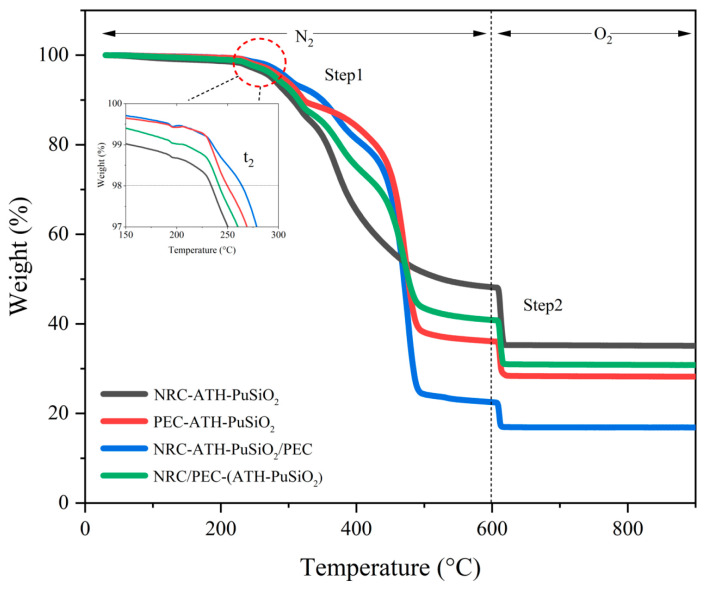
TGA thermograms of NRC and PEC filled with ATH and PuSiO_2_ under different mixing processes.

Figure 6 shows the TGA thermogram where it is possible to evaluate the thermal degradation of the composites and blends decomposed under N_2_ and O_2_ atmospheres to measure the resistance to thermo-oxidative degradation of the NRC and PEC after being combined with several components. It is noted that the 1% difference at temperatures below 150 °C is due to the evaporation of small moisture during the increasing temperature. A weight loss attributed to the decomposition of the segments of PEC occurred between 180 and 330 °C. This is also the overlapping degradation with the PU hard segment which degraded at approximately 260 °C. In the second stage, a weight loss was observed at 302–433 °C, indicating the degradation of the NRC when heated. In the third stage, at 425–520 °C, the weight loss was attributed to the thermal degradation of the PE and NR chains. Additionally, the onset temperature (*t*_2_) increased when PE was added to the blend, indicating increased crystallinity or hardness in the bulk composite. However, there was a significant increase in residual weight in the formulations containing NR, indicating several impurities in NRC, such as SiO_2_, ZnO, and sulfur compound. Considering the degree of decomposition under heating in the TGA, the PEC decomposed at temperatures above *t*_2,_ as can be seen in the case of the incompatible PEC and NRC (NRC-ATH-PuSiO_2_/PEC), which showed the highest degree of decomposition. At this stage, the NR showed its potential to resist deformation and degradation; therefore, NRC-ATH-PuSiO_2_ exhibited a lower value. With this same rationale regarding the effect of reinforcing the main matrix, NRC/PEC-(ATH-PuSiO_2_) showed better thermal properties than the other composites that contained PEC. Thus, a decrease in decomposition in the first step led to a higher degree of decomposition in the second step, as seen in Figure 6 and Table 4.

### 4.6. UL-94 Flammability

**Figure 7:**
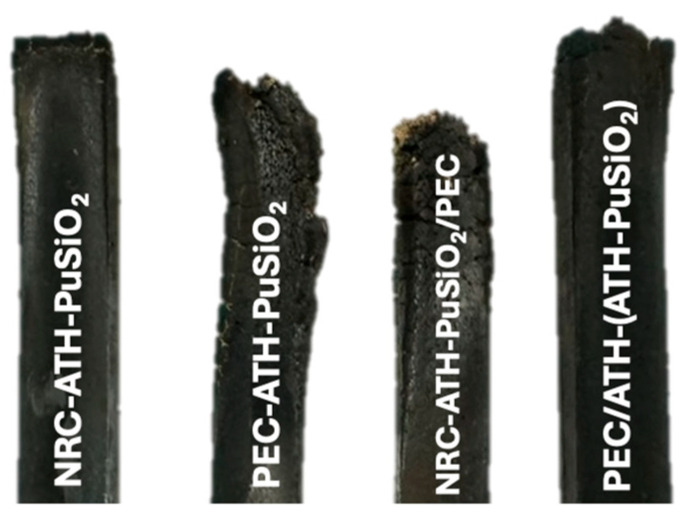
Physical appearances of NRC and PEC filled with ATH and PuSiO_2_ after flammability tests.

From the UL-94 tests of NRC-ATH-PuSiO_2_, PEC-ATH-PuSiO_2_, NRC-ATH-PuSiO_2_/PEC, and NRC/PEC-(ATH-PuSiO_2_), as shown the received results in Figure 7 for the physical appearances and Table 5 as the flamability stadard, NRC-ATH-PuSiO_2_ and NRC/PEC-(ATH-PuSiO_2_) passed the test criteria with a V-0 rating. This indicates that the sample exhibits efficient flame retardancy properties with good dispersion and distribution of the ATH inside both bulk polymeric matrixes. A high shear force during mixing also caused homogenous dispersion of the PU waste and SiO_2_ particles throughout the NRC and PEC matrixes. Here, the PU and SiO_2_ synergistic filler forms protective flame expansion layers, delaying the spread of flames. In addition, the ATH released water during burning propagation, as seen in the model proposed in Figure 6. This released water helps dilute the flames and increase combustion, producing better flame retardancy [44]. However, when the NR composites were mixed with incompatible pure PE in a 50:50 ratio, it achieved a V-2 rating, possibly because PE is a flammable material that is easily ignited.

## 5. Conclusions

NRC and PEC composites filled with ATH and PuSiO_2_ under different mixing processes were prepared using an internal mixer and a two-roll mill. NRC and PEC filled with PU and SiO_2_ at a total concentration of 300 phr with ATH at 140 phr were investigated. The PEC-ATH-PuSiO_2_ and NRC/PEC-(ATH-PuSiO_2_) showed the highest modulus and tensile strength due to the crystallinity of the short-chain hydrocarbon molecules. This also caused the improved initial storage modulus and *Tan δ* at 0 °C of the proposed composite and blend. For NRC-ATH-PuSiO_2_ and NRC/PEC-(ATH-PuSiO_2_), NRC provided a higher elasticity by increasing the elongation at break with improved impact strength and shifting *T_g_*. Due to the low degree of decomposition of NRC, the NRC/PEC blend also had low decomposition with increased temperature. Therefore, with the dispersion and distribution of the ATH, PU waste, and SiO_2_ throughout the NRC and NRC/PEC matrixes, V-0 levels were assigned, and these blends are highly flame-retardant. Thus, there are two possible applications for providing superior stiffness and high elasticity products for both indoor and outdoor applications.

## Figures and Tables

**Table 1:** Formulation of NRC and PEC filled with ATH and PuSiO_2_ under different mixing processes.

Chemicals	NRC-ATH-PuSiO_2_	PEC-ATH-PuSiO_2_	NRC-ATH-PuSiO_2_/PEC	NRC/PEC-(ATH-PuSiO_2_)
**Pure PE existing**	**0 phr**	**100 phr**	**50 phr**	**10 phr**
	**Part 1 (phr)**			
Pure NR	100	-	-	-
Pure PE	-	100	-	-
PU waste	200	200	-	-
SiO_2_	100	100	-	-
ATH	140	140	-	-
Steric acid	1	-	-	-
ZnO	5	-	-	-
TMQ	1	-	-	-
MBTs	1	-	-	-
Sulfur	2	-	-	-
	**Part 2 (phr)**			
Pure PE	-	-	50	-
PEC-ATH-SiO_2_	-	-	-	50
NRC-ATH-SiO_2_	-	-	50	50

**Table 2:** Summary of Young’s modulus, tensile strength, elongation at break, and hardness of NRC and PEC filled with ATH and PuSiO_2_ under different mixing processes.

Samples	Young’s Modulus (MPa)	Tensile Strength (MPa)	Elongation at Break (%)	Hardness (Shore A)
NRC-ATH-PuSiO_2_	1.54 ± 0.15	5.87 ± 0.28	14.00 ± 1.01	94.5 ± 1.04
PEC-ATH-PuSiO_2_	3.84 ± 0.09	8.02 ± 0.42	2.96 ± 0.22	97.0 ± 2.07
NRC-ATH-PuSiO_2_/PEC	2.59 ± 0.17	3.30 ± 0.73	2.89 ± 0.68	84.0 ± 0.50
NRC/PEC-(ATH-PuSiO_2_)	2.96 ± 0.16	7.45 ± 0.46	5.22 ± 0.56	96.0 ± 0.35

**Table 3:** Initial *E’*, *T_g1_*, *T_g2_*, and *Tan δ* at 0 °C of NRC and PEC filled with ATH and PuSiO_2_ under different mixing processes.

Samples	Initial *E’* (MPa)	*T_g1_* (°C)	*T_g2_* (°C)	*Tan δ* at 0 °C
NRC-ATH-PuSiO_2_	1787	-	−52	0.09
PEC-ATH-PuSiO_2_	2071	−129	-	0.06
NRC-ATH-PuSiO_2_/PEC	1514	−127	−54	0.09
NRC/PEC-(ATH-PuSiO_2_)	2024	−126	−56	0.07

**Table 4:** Decomposition degree and the onset temperature of NRC and PEC filled with ATH and PuSiO_2_ under different mixing processes.

Samples	Decomposition (%)		Temperature (°C)
	First Step	Second Step	t_2_
NRC-ATH-PuSiO_2_	50	13	238
PEC-ATH-PuSiO_2_	63	8	254
NRC-ATH-PuSiO_2_/PEC	76	6	268
NRC/PEC-(ATH-PuSiO_2_)	58	10	245

**Table 5:** UL-94 flammability of NRC and PEC filled with ATH and PuSiO_2_ under different mixing processes.

Samples	Fire Classification
NRC-ATH-PuSiO_2_	V-0
PEC-ATH-PuSiO_2_	V-2
NRC-ATH-PuSiO_2_/PEC	V-2
NRC/PEC-(ATH-PuSiO_2_)	V-0

## Data Availability

The raw data supporting the conclusions of this article will be made available by the authors on request.
